# Risk Assessment in Drug Hypersensitivity: Detecting Small Molecules Which Outsmart the Immune System

**DOI:** 10.3389/falgy.2022.827893

**Published:** 2022-02-22

**Authors:** Werner J. Pichler, Stephen Watkins, Daniel Yerly

**Affiliations:** ADR-AC, Bern, Switzerland

**Keywords:** drug hypersensitivity—prevention and control, p-i concept, fake antigen, HLA, structural analysis, T cell receptor for antigen, risk assessment

## Abstract

Drug hypersensitivity (DH) reactions are clinically unusual because the underlying immune stimulations are not antigen-driven, but due to non-covalent drug-protein binding. The drugs may bind to immune receptors like HLA or TCR which elicits a strong T cell reaction (p-i concept), the binding may enhance the affinity of antibodies (enhanced affinity model), or drug binding may occur on soluble proteins which imitate a true antigen (fake antigen model). These novel models of DH could have a major impact on how to perform risk assessments in drug development. Herein, we discuss the difficulties of detecting such non-covalent, labile and reversible, but immunologically relevant drug-protein interactions early on in drug development. The enormous diversity of the immune system, varying interactions, and heterogeneous functional consequences make it to a challenging task. We propose that a realistic approach to detect clinically relevant non-covalent drug interactions for a new drug could be based on a combination of *in vitro* cell culture assays (using a panel of HLA typed donor cells) and functional analyses, supplemented by structural analysis (*computational data*) of the reactive cells/molecules. When drug-reactive cells/molecules with functional impact are detected in these risk assessments, a close clinical monitoring of the drug may reveal the true incidence of DH, as suppressing but also enhancing factors occurring *in vivo* can influence the clinical manifestation of a DH.

## Introduction

Drug hypersensitivity (DH) represents a substantial problem for certain drug classes such as beta-lactams, sulfanilamides and other antibiotics, anti-epileptics, radiocontrast media, and muscle relaxants (1). The symptoms of DH range from harmless exanthema to deadly anaphylaxis or Stevens Johnson syndrome (SJS)/toxic epidermal necrolysis (TEN). Some reactions are local, some systemic, some appear within minutes, and some after weeks. The underlying mechanisms are heterogeneous and diagnosis is cumbersome. Essentially, it is an iatrogenic disease, making DH a challenging medical condition.

For decades DH was explained by the hapten concept. This was developed more than 90 years ago by K Landsteiner and others, stating that small molecules like drugs or other chemicals are too small to function as an antigen for the immune system (2). Only if the drug acts as a “hapten,” meaning that it binds via covalent bonds *stably* to a protein and thus forms a larger drug-protein adduct, it functions as an antigen to which immune reactions may develop (3). An immune response develops if co-stimulation is provided, e.g., by some “toxic” effect of the drug (contact dermatitis model) (4).

This hapten concept has been well-established in immunology and has been validated by animal and *in vitro* experiments which have confirmed hapten-specific antibody and T-cell responses (3–6). The immune reactions to the drug-protein adducts include productive immunity (T cells, antibodies) or tolerance (induction of Tregs) (6, 7). When only the antigen without co-stimulation is provided, non-responsiveness may ensue (8). Thus, an essential, although not sufficient step in initiating immunity to small molecules appears to be antigen formation generated following a covalent link between the small compound and the protein.

However, various clinical and research findings have challenged an exclusive role of hapten-formation in DH (9–11). Alternative routes of immune stimulation by drugs have been proposed which may better explain the sometimes self-destructive nature of the clinical reactions in DH [summarized in Pichler (12)].

**Figure 1:**
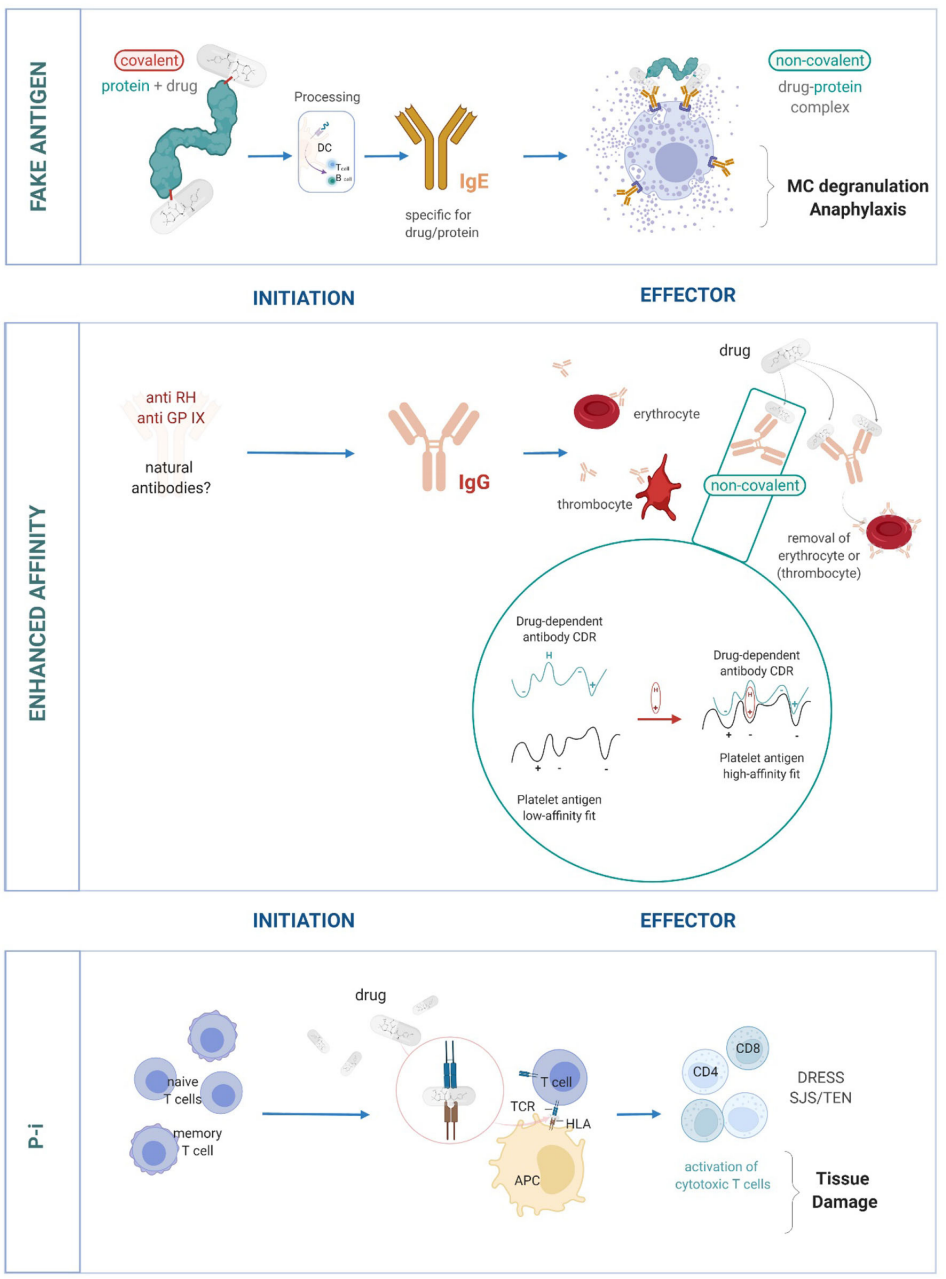
The important role of non-covalent drug-protein interactions in eliciting DH. Three models of DH reactions: Fake antigen: the non-covalent drug-protein complexes imitates the true antigen complex (based on covalent bonds). The formation of fake antigens is quick and occurs in high quantities. It can interact and cross-link preformed drug-specific IgE and thus can overcome mast cell unresponsiveness (11). Enhanced affinity: antibodies with low affinity to cell surface proteins on blood cells are already present. A drug binds to such antibodies and thereby increases their affinity to the target structure. This can result in the elimination of blood cells (thrombocytes and erythrocytes) (10, 13–15). Pharmacological interaction of drugs with immune receptors like HLA and TCR (p-i). The drug binds to HLA, which makes these surface structures look like an allo-HLA allele and elicits a strong T-cell response. Binding to TCR stimulates in a similar allo/superantigen-like way. The stimulated T cells are polyspecific, cytotoxic and various late effects may occur (12, 16, 17).

In brief, these unusual stimulations comprise (Figure 1):

- the *p-i concept*, where drugs bind to immune receptors such as the human leukocyte antigen (HLA) or T cell receptors (TCR) and induce an allo-like immune reaction (9, 16, 18–20). These reactions would explain the majority of delayed systemic DH.
- the *enhanced affinity model*, where drugs bind to pre-existing complementarity-determining regions (CDRs) of antibodies which bind surface structures on thrombocytes or erythrocytes. This enhances their affinity and effects, which may lead to drug-induced antibody-mediated blood cell dyscrasias (10, 13–15).
- the formation of a *fake antigen*, where the drug binds to soluble proteins like albumin, which can then cross-link preformed, surface-bound, drug-specific immunoglobin (Ig)E on mast cells causing anaphylaxis/acute urticaria (11). This is a recently proposed model and provides further explanation into DH reactions.

All three types of DH are due to the non-covalent binding of drugs to proteins. Such reactions are normally ignored by the immune system. However, immune stimulations may occur when two parameters are fulfilled. First, the type of targeted protein is an immune receptor. It can be the TCR or the CDR region of antibodies, which may be directly involved in signaling and effector functions of the specific immune system. If the drug binds to the HLA-peptide complex, a T cell reaction is evoked, which imitates an allo-like stimulation (12, 16). Second, the sum of various non-covalent drug-interactions may allow a sufficiently affine drug-protein binding, which in turn stimulates an immune reaction by reactive T cells or other effector functions. These concepts and models have been extensively reviewed by ourselves and others (10, 12, 16, 21).

However, assessing risk for DH during drug development has always been difficult and there are no suitable animal models to predict DH. Therefore, confusion can ensue when a drug induces potential DH reactions during clinical trials. For example, one drug, which was not acting as a hapten pre-clinically, could elicit a potentially immune-mediated adverse reaction. Drug development was then stopped, the side effects were not analyzed or investigated further, and the opportunity to establish whether this was due to DH and the condition for the DH manifestation, was missed. Therefore, assessing the risk of DH whilst generating novel drugs and exploring DH as the cause of adverse reactions in patients is essential to enhance our understanding of DH and the off-target effects of novel drugs.

Here we review the current knowledge regarding the risk assessment of DH and discuss further considerations for the field.

## How Relevant is Non-Covalent vs. Covalent Drug Protein Binding in DH?

While the existence of DH due to non-covalent binding is well-accepted and patients and symptoms have been extensively described, the question is open, how frequent are non-covalent drug-protein bindings for DH: Is only a minority of DH caused by non-covalent binding, or is it the majority?

It is difficult to determine what kind of drug-protein bindings takes place in each case of DH (21): therefore one extrapolates from the investigation of a few patients to all, provided the drug or HLA is the same: e.g., if carbamazepine is found to bind to HLA-B^*^15:02 in SJS—one generalizes this finding to all SJS after CBZ in B^*^15:02. Similar data are available for a panel of drugs and DH-manifestations (22, 23). Since the data also show, that, when analyzed, binding was non-covalent, we postulate that p-i reactions are frequent and represent the *dominant* form of T-cell mediated DH. This conclusion is based on following observations:

- p-i stimulations comprise DH which show an HLA-association, since the binding of a drug to a certain HLA-allele—which is the cause for this link—is non-covalent (24–29). The list of such drugs is long (30);
- The immunological (16) and clinical features of DRESS and SJS/TEN (with and without HLA association) are suggestive for p-i, and where investigated, a p-i mechanism was found (30, 31). Thus, the majority of severe T cell mediated DH are due to p-i.
- The situation is less clear with maculopapular exanthems (MPE): these milder reactions have often no clear HLA-link: This, because the drug may bind with lower affinity to various HLA-alleles. Nevertheless, where investigated, they were p-i (32, 33).
- Pustular exanthems appear to have different origins: Some may appear together with DRESS or exanthems and involve p-i mediated T cell stimulations (34). But other forms of AGEP appear more rapidly (<4 days) and may be due to other mechanism (35).

The enhanced affinity model does also seem to be the most frequent in blood cell dyscrasias (10, 13–15). Interestingly, even when a drug which has the ability to bind by covalent means to its target protein (e.g., beta-lactams), the cause blood cell dyscrasias were often due to non-covalent bindings as revealed by the lability of binding (36).

The frequency for the fake antigen model is unknown. However, it is—at present—the most likely explanation for immediate (<10 min), IgE mediated degranulation of mast cells by a drug-protein complex; The formation of covalent binding take longer than the seconds/few minutes which pass between drug exposure and start of anaphylaxis symptoms; For some drugs (e.g., sulfamethoxazole) the drug must first be metabolized into a reactive compound before it could bind (3, 18, 19); this would require hours, but symptoms occur within minutes (11).

## Changing The Rules For Risk Assessment Of Systemic DH

### Principles of Risk Assessment for DH

Risk assessment in immune-mediated adverse drug reactions (ADR) requires an understanding of how the immune system is initially stimulated by a drug, and how the effector functions are elicited. Questions to consider include:

### Is the Drug Able to Form an Antigen?

If yes, then it could elicit the whole spectrum of immune reactions, namely antibody and T cell reactions (Gell & Coombs I-IV) if some co-stimulation is provided (3–5, 16, 17). This was a key concept in the longstanding risk assessment for DH. Focussing exclusively on hapten/covalent bindings and the subsequent immune reactions to it, ignored severe immune-mediated reactions that occurred mainly after exposure to non-hapten drugs (16).

### Is DH the Consequence of an *Abnormal* Immune Stimulation by Non-covalent Interactions?

**Figure 2:**
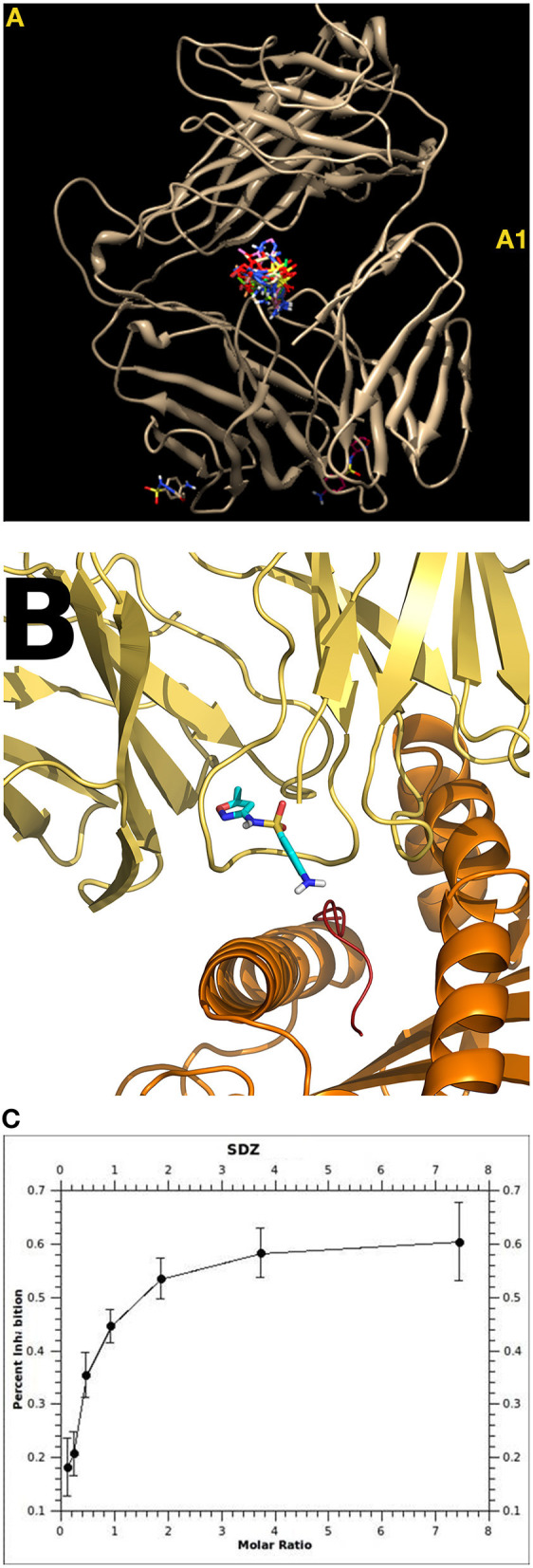
Interaction of sulfamethoxazole (SMX) with TCR. Shown are details of the specificity at the molecular level and the influence of drug orientation. The position in TCR signaling can differ from only a few atoms between similar drugs, highlighting the sensitivity of the immune interactions (39). **(A)** Sulfanilamide may bind to different regions of a TCR. Some of these binding sites were common/public **(A1)**—and its functional consequence is unclear. Other binding sites were unique for the SMX-specific TCR: TCR “H13” showed strong binding of SMX and 5 stimulatory compounds to the CDR2 (Vβ) [details see (37)]. The TCR “1.3” contained a prominent CDR loop of Vα, where all sulfanilamides tested bound (38). **(B)** Docking (autoDock and AutoDock Vina Software) of SMX and 11 other sulfanilamides showed a unique site in the CDR3 loop on TCR 1.3. The other 11 sulfanilamides bound as well: docking revealed that only SMX were able to bind in a functional orientation, allowing the NH2 end to interact with the HLA presented peptide. All other compounds tested were able to block SMX induced activation (Ca++ influx and proliferation) (38). **(C)** Inhibition of SMX induced proliferation by the non-stimulatory sulfanilamides; shown is the inhibition of SMX stimulation by SDZ (Sufadiazine); the stimulation of TCC 1.3 was measured by 3-H thymidine incorporation. Eleven sulfanilamides inhibited in a dose dependent way (ratio SMX: sulfanilamide: 1:8–8:1); the maximal inhibition was ca. 60–80%; all assays were done in non-toxic concentrations (below 250 microg/ml) [details see (38)].

If yes, then one has to consider the following points:

i. *The non-covalent drug-protein interactions are labile and reversible* but still strong enough to occasionally transmit signals. Additional factors, like generalized T cell activation due to viral infections for example, may influence whether these non-covalent interactions effect function, or result in DH (16). Thus, a clinical evaluation is necessary to obtain the complete picture.
ii. *P-i stimulations follow the rules of pharmacological interactions*. The binding of the drug results in signaling, partial signaling, or no signaling. The signal could be blocked by competitive drugs, could be influenced by the orientation of the drug-binding, or could elicit an allosteric effect (37, 38) (Figure 2). Thus, the possible consequences of drug binding seem to exceed antigen-immune receptor interactions as observed in protein/peptide recognition.
iii. The *clinical symptoms fall outside the normal*, mostly focused and local immune stimulation. In contrast to antigen stimulation, where immune-regulatory mechanisms prevent inappropriate immune stimulations, uncommon and generalized symptoms to the unorthodox immune stimulation may appear (11).

Judging by the list itself, it becomes evident that it is not easy to include all these variables in a risk assessment for DH, mainly due to the seemingly unstable bindings.

Besides the above-mentioned points to consider, risk assessment for DH should also include the questions shown in Table 1.

**Table 1:** Demonstration of immune stimulatory potential of a new drug.

**•Hapten feature of a drug/metabolite (or not)? •p-i stimulation of T cells (or not)? •If a reaction occurs: phenotype and function, HLA/TCR restriction, crossreactivity; •docking data of the involved molecules/cells, best in combination with functional analysis: where does it bind? •Non-covalent binding to Ig specific for blood cell surface molecules? Has this a clinical effect? •If drug specific IgE was generated, can the Fc-IgE-RI bound IgE be cross-linked by non-covalent drug-protein complexes? •Clinical evaluation/monitoring of ADR and possible manifestations of ADR dependent on co-stimulation or tolerance**

### Risk Assessment and Hapten Mechanism

Risk assessment for DH has recently been extensively reviewed by Hammond et al. (40). They describe different *in silico* and *in vitro* approaches in detail, including hapten and non-hapten drugs (see Box 1).

Box 1 **Background/Terminology.**

**Hapten:** Haptens are small molecules that elicit an immune response when bound stably (covalently) to a carrier protein.

**Covalent bond:** a chemical bond that involves the sharing of electron pairs between atoms. The hapten/drug-protein complex is called an adduct.

**Non-covalent bindings** involve various electromagnetic interactions between molecules or within a molecule. Non-covalent interactions involve electrostatic, π-effects, van der Waals forces, and hydrophobic effects. They are labile and reversible, but the sum of non-covalent forces can be substantial.

**Antigen processing and drug-protein complexes:** The drug-protein complex based on non-covalent bindings is disrupted by antigen processing in antigen processing cells (e.g., dendritic cells), which prevents formation of a new antigen for T cells: such labile complexes are unable to initiate an complete immune response. In contrast, the covalent bond between hapten/drug-protein is persisting antigen processing, and an immune reaction (which requires co-stimulation) to hapten-protein or hapten-peptide complexes can evolve.

**Contact dermatitis:** Application of haptens onto the skin can elicit a sensitization of T cells (CD4 and CD8) and a contact dermatitis, which is an inflammatory skin disease. Many of the contact allergens have also an irritating (toxic) effect (danger signal), which leads to activation of dendritic cells and thus provides co-stimulation. Thus, haptens have the ability to form antigens (drug-protein adducts) and may act co-stimulatory.

**Allo-immune stimulation:** The maturation process in the thymus leads to T cells in the circulation which react to foreign antigenic peptides in the context of self HLA molecules (HLA-restriction). But at least 10–25% of circulating T cells do directly react with certain foreign HLA molecules with peptides (=allo-HLA) as well. This phenomenon is called *direct* allo-recognition: it is a strong T cell activation and explains the acute transplant rejection and it‘s *in vitro* correlate, the MLR (mixed leukocyte reaction). The p-i stimulation is thought to imitate such an allo-immune reaction, and MPE, DRESS as well as SJS/TEN are thought to be the result of such aberrant immune stimulations with emphasis on proliferative CD4 and CD8 stimulations (MPE, DRESS) or mainly CD8/NK cell activations (SJS/TEN).

Proving the hapten features of a drug or drug metabolite has been the cornerstone of risk assessment for DH for years, and the formation of an antigen was considered essential. Many data on hapten features originate from contact dermatitis research, and reliable *in vitro* methods to identify the hapten characteristics of a drug have been elaborated upon (41, 42). The existence of a potential hapten feature of a systemically applied drug remains to be determined for contact dermatitis, and the fake-antigen model alike. In the latter, the original IgE might have been developed against the (covalent) drug-protein complex (the “true” antigen), whilst the fake antigen interacts with these preformed antibodies and causes degranulation (11).

Of note, hapten-protein adducts can induce complete immune reactions, including antibodies. But these are, even if IgE is involved, not automatically harmful, as long as the they form slowly and in low quantities (11). It has been suggested that symptoms of anaphylaxis appear only if the matching antibody can be stimulated by a rapidly formed fake antigen in large quantities (11). Consequently, when hapten and IgE generation are detected, an analysis of fake antigen formation would be required.

The enhanced affinity model also relies on preformed antibodies. However, here the specificity of the antibody is not directed to the drug-hapten adduct, but to surface structures of blood cells. Such antibodies may be modified by non-covalent drug interactions, leading to an enhanced affinity for the target structure (Figure 1). Therefore, screening for a hapten characteristic of these drugs is not constructive for such a mechanism.

Conversely, antigen formation is not required for T cell reactions following p-i (HLA, TCR). The occasional large magnitude T cell stimulations occur due to the allo-like nature of the p-i modification on the TCR/HLA complex (12, 16, 17, 43). Thus, the demonstration that a drug can act as a hapten and form hapten-protein adducts is relevant in contact dermatitis and the fake antigen model, but its role in other models of DH is unclear and possibly irrelevant.

### Risk Assessment of p-i Stimulation of T Cells: Cell Culture Assays

The task of risk assessing of a new drug would be to detect relevant (stimulatory) drug binding due to non-covalent interactions with immune receptors like HLA and/or TCR. It has repeatedly been shown that human blood-derived PBMCs from drug naïve donors following a 2–6 weeks drug exposure can, under optimal conditions, result in outgrowth of drug-reactive T cell lines originating from the memory and naïve T cell pool (43, 44). This stimulation occurs with both hapten-like and non-hapten drugs (p-i). It is cumbersome and requires optimal cell culture conditions for T cell expansion, such as the addition of IL-2 and feeder cells (45–49).

The feasibility of this approach using drug-naive individuals was originally assessed using cell cultures stimulated with sulfamethoxazole, flucloxacillin, abacavir, allopurinol, oxypurinol, and antiepileptics and relied mostly on known blood donors expressing the risk allele (45–49). The outgrowth of drug reactive cells could be detected after 2–6 weeks by measuring cytokine secretion, upregulation of activation markers, or proliferation. Notably, similar effects could also be induced in cell cultures not expressing the risk allele, as the drug was bound to various HLA-proteins or TCR (46, 47, 49). Under these conditions, the outgrowth of cells seemed to require more time compared to cell cultures expressing the risk allele.

The immune receptor molecules are extremely heterogeneous, both in a population, as well as in individuals (with estimated >10^7^ TCR). It is the highly diverse antigen-binding site of TCR or the peptide binding region of HLA where functionally relevant drug interactions may occur (22–26, 37, 38). Thus, only a few T cells may react to a single drug, and the outgrowth of such drug-reactive T cells requires time (weeks). If the specific binding molecules (e.g., an HLA-allele) are not included in the cell donor panel, one might miss a potential reactivity. Since one individual expresses >10^7^ TCR but only 7–14 different HLA-alleles (of >10^5^), one has to analyze >100 individuals with defined HLA to cover at least a substantial different part of HLA-alleles (40).

A research group from Liverpool intensively investigated the cell culture approach using a panel of HLA typed donors (40, 48, 49). They concluded, that a negative cell culture could mean a true lack of stimulation, an absence of presenting HLA in the selected blood donors, or unsuccessful stimulations (due to the experimental conditions). To enhance the detectability of the stimulation, certain modifications were proposed. It was proposed that dendritic cell (DC) stimulation or DC-enrichment might enhance reactivity (40). However, our data show that stimulation is possible without the involvement of DCs (43). Since certain HLA alleles are more often implicated in p-i HLA based T cell stimulations, blood donors that carry risk alleles (e.g., the B^*^15:02, B^*^57:01, or B^*^58:01) which are more commonly involved in DH can be selected (22–24). Also, the ethnicity of blood donors should be considered. The HLA-B^*^15:02 is important for carbamazepine-induced SJS in Southeast Asia, but is practically absent in blood donors of European origin. Thus, would have been missed using an only European cell panel. Of interest would be to investigate blood from donors, which previously had multiple drug hypersensitivity syndromes (MDH), as it should react to multiple drugs also *in vitro* (50).

### Risk Assessment of p-i Stimulation of T Cells: Characterizing the Reaction

If drug stimulation is successful, the outgrowth of T cells can be further analyzed to define the drug-presenting HLA-allele, the TCR involved, cytokine production, cytotoxicity, and cross-reactivity. Such analyses in combination with docking studies may identify the potential drug binding site on the immune receptor (HLA or TCR), the affinity, and whether drug binding occurs in a functionally relevant location.

There are already some data available on p-i HLA or p-i TCR (37–39, 51). Over the years, a panel of TCR-HLA pairs involved in cell stimulation by different drugs, was obtained and partly characterized. Some of these unique drug reactive TCR were transfected into various permanently growing cell lines (52–54). A list of such TCR transfected cell lines mostly derived from DH patients is given in Table 2. Their further molecular analysis might be helpful to better understand the molecular interactions and define drug bindings as relevant.

**Table 2:** List of cell lines transfected with drug-reactive TCR.

**Drug**	**TCR**	**TRBV**	**TRAV**	**HLA restriction**	**Note**	**source**	**References**
Sulfamethoxazol	UNO-H1.3	20-1	17	DR-B1^*^10:01	CD4, wide CR	Patient, MPE, malaise, hepatitis	(53)
Norfloxacin	Ruba1	5-4	5	MHC-II	CD4 selective CR	Patient, MPE	(53)
Ciprofloxacin	Ruba2	20-1	26-1	MHC-II	CD4 selective CR	Patient, MPE	(53)
Abacavir	BeS-B7	2	19-5	B^*^57:01	allo B^*^58:01 and peptide	Unexposed	(55)
Abacavir	MiLu 2D	20	12	B^*^57:01	CD8	Unexposed	(55)
Abacavir	MiLu 17D	05	9-2	B^*^57:01	CD8	Unexposed	Unpublished
Abacavir	UL3L	7-2	26-1	B^*^57:01	allo B^*^58:01	Unexposed	(55)
Allopurinol	AnWe	12-4	13-2	A^*^33	CD8	Patient, SJS	Unpublished
Oxypurinol	SeTr	25-01	11-2	B^*^58:01	CD8	Patient, SJS	(56)
Flucloxacillin	MarGa1	6-5	21	B^*^57:01	CD8	Unexposed	Unpublished
Iomeprol	T5227/iom28	6-6	22-1	MHC I	CD8, no CR	patient, MPE	(54)

*CR, crossreactivity*.

The crystal structure of abacavir in complex with the drug-binding pocket of HLA-B^*^57:01 unveiled a new mechanism for drug-specific T cell stimulation by altering the peptide presentation of drugs that can bind to the HLA inside the endoplasmic reticulum and replace the normally presented peptide (55–57). Therefore, molecular modeling and crystallography, in combination with functional assays, is a powerful tool to study DH reactions (37, 38, 51, 58).

The p-i TCR stimulations are less studied compared to p-i HLA. However, studies investigating these interactions indicated that these stimulations followed the rules of classical pharmacology e.g., drug-receptor interactions, but did not depend on antigen recognition: Various rules of T cell immunology were ignored. There was no HLA restriction, the immunogenic peptide was exchangeable, and the T cells showed high allorecognition (16, 17).

Two sulfamethoxazole (SMX) specific T cell clones (TCC 1.3 and H13) were investigated in detail (37, 38) (Figure 2). They originated from the same patient with DH (exanthema, hepatitis, nausea) to SMX. In H13 it could be shown with molecular modeling that the SMX binding exerted an allosteric effect on the configuration of the TCR, which bound 7-fold more affine to the HLA-peptide complex than the drug (37).

The TCR 1.3 bound SMX without via a dominant loop in the TCR (TCRVα3) (Figure 2) (38). Ca^2+^-influx to SMX occurred as early as 14 s after adding SMX to the TCC 1.3 and APC. Interestingly: the analysis revealed blocking of SMX stimulation by related, but not activating sulfanilamides, which could be traced back to the orientation of the sulfanilamide binding to the TCR (Figure 2B). Thus, even just analyzing two TCC from one individual by combining functional and computational data (e.g., using autoDock and AutoDock Vina Software) unveils large complexity of the drug effects toward TCR. It would not be surprising if we detected additional mechanisms contributing to T cell activation if more interactions were analyzed in detail.

### *In vitro* Reactivity ≠ Drug Hypersensitivity *in vivo*

Computational and *in vitro* data only suggest that an immune reaction might be possible, they do not prove that a drug elicits a DH reaction. In reality, the *in vitro* data overestimates the DH risk. Regulatory mechanisms *in vivo* appear to suppress most of these reactions (59). For example, abacavir in B^*^57:01+ carriers elicited a reaction *in vitro* in 100% of the tested samples but occurred *in vivo* in only 53% (60, 61). Other drugs have a far lower penetrance (ca. 3% in CBZ and B^*^15:02) but regularly show a reaction *in vitro*.

At present, the details and extent of DH-suppressing regulatory mechanisms are unknown. *In vitro* risk assessment can deliver a signal, but the value of this signal needs to be verified by *in vivo* testing. Rigorous post-marketing surveillance is required and is an important tool to detect and differentiate true DH in relation to the immunological risk revealed by *in vitro* tests. It is also important for monitoring other risk factors that develop only in *in vivo* setting such as cases where a DH only manifests when the patient is exposed to a drug during an infection which causes massive immune stimulation (HIV, CMV, or EBV) (16).

Animal experiments have not been successful in predicting DH. However, models for DH using HLA and TCR transgenic mice have been recently developed (51, 59). These models are an important extension of the *in vitro* data and are ideal to understand the mechanisms of this particular DH and symptoms more in detail. But one or more animal models cannot grasp the vast possibilities of an endless number of drugs and their interaction with highly diverse immune receptors in the population.

### Increased Affinity Model

When drug binding can increase the antibody affinity for the target structure and the reactive antibodies are already pre-formed, this can cause blood cell dyscrasia as a form of DH (Figure 1). For example, the antibodies in drug-induced immune thrombocytopenia are directed against platelet membrane glycoprotein IX or in hemolytic anemia against Rh blood cell surface sugars (10, 13–16). They are not drug-specific and the origin of these antibodies is unclear. They may be natural antibodies. Models of these are easy to construct simply from genetic data already available. These can serve as a basis for initial drug-Ig interaction *in silico* and further tests *in vitro*.

To optimize the risk assessment for such DH, one might also learn from previous patients and isolate and determine their antibodies. The drug-binding sites could be identified and models with the contact region of cell surface molecules could be generated. Based on these models a database of these antibody structures with binding sites could be established and be applied for drugs in development.

### Fake Antigen Model

In the fake antigen model, the drug-reactive IgE may have been generated to the covalent drug-protein complex e.g., a beta-lactam binds to accessible peptide regions in the protein which carries a lysine group. This binding occurs first by non-covalent means, then by covalent bonds. This adduct formation is slow and stable and allows the generation of IgE antibodies to this drug protein adduct (11).

To form a fake antigen, the same beta-lactams bind to the same location, but only via non-covalent interactions. This interaction is fast (seconds to minutes) and can already be sufficiently affine to cross-link the previously formed IgE to beta-lactams, causing degranulation.

The difficulty of risk assessment in the fake antigen model is that many anaphylactic reactions presumably based on the fake antigen concept occur during the first encounter with the drug. To evoke a response, the drug reactive IgE must have been developed beforehand. But how can this be explained if the patient was not (knowingly) exposed to the drug before? We hypothesize it must be an antigen which is able to elicit IgE, but also able to react with the fake-antigen (non-covalent drug-protein complex). It is even possible that a completely unrelated structure elicits IgE antibodies, which cross-reacts with the drug-protein complex (a type of heterologous immunity). Fake antigen formation is a new concept and has not yet been considered in risk assessment but must certainly be considered. More data are needed to further develop or explain this model.

## Conclusion

It is a challenge to address the risk assessment in DH, which is defined by pharmacologists as a “non-predictable,” Type B ADR (62). But by challenging the hapten model as the sole explanation for DH, a first step to improve the situation may have been made. We may now be closer to understanding these interactions, but the explanations are still complicated.

Non-covalent drug-protein binding is normally unstable, transient, and ignored by the immune system. The challenging task of risk assessing severe DH would be to detect these rather transient drug-protein bindings as a potential cause for DH.

We propose to combine

a) *in vitro* data (human cell cultures with drugs, defining drug interaction with different HLA-alleles, TCR, Ig structures, generation of drug-specific T-cell lines, functional analysis) with
b) computer-based docking data (drug interaction with HLA, TCR, Ig) and
c) clinical surveillance for risk assessment.

Common animal experiments are not helpful, as interactions causing adverse reactions are due to very specific, human immune receptors. There may be a limited role for gene-modified animals (expressing human HLA, human TCR (51, 59), but mainly for specific questions to proof a concept but not for risk assessment in general.

The risk assessment focussing on non-covalent drug-protein interactions is in its infancy. Investigating patients with prior DH and MDH may deliver insights into how drugs induce immune stimulations in real life. Molecular analysis of drug activation using TCR transfected cell lines (Table 2) may provide insights into common risk areas for drug binding and help to discriminate relevant from non-relevant drug-protein interactions in computer models.

Accurate risk assessment should be based on openly sharing of data of ADRs and investigational research by pharmaceutical companies, a practice which is not fully embraced. Moreover, it is important that academia and pharmaceutical industries combine their efforts to establish a database of clinical DH data and corresponding TCR, HLA, Ig, and of the involved drugs. Finally, not only data, but the courage to challenge and replace the outdated approach for DH risk assessment, is required.

One lesson learned from previous DH with defined risk factors is that the *in vitro* risk assessment overestimates the *in vivo* risk (60, 63). Regulatory mechanisms may prevent manifestations of clinical DHs (59), or cofactors which may enhance DHs (16), are not known and cannot be considered in *in vitro* models. A possible presentation of DH should be considered during surveillance of the drug and one should be prepared to perform immunological analysis and carefule documentation of the affected patients, if a side effect appears.

It is more satisfying to conduct research on the beneficial, rather than detrimental consequences, of a drug, like ADR. However, the great success of personalized medicine in DH prevention by identifying risk alleles, is allowing prediction of the unpredictable ADR (60, 63). Thus, in the future the analysis of a new drug should also include assessment of off-target effects of a drug, which includes potential interactions with highly polymorphous immune receptors.

## Data Availability Statement

The original contributions presented in the study are included in the article, further inquiries can be directed to the corresponding author.

## Author Contributions

WP: design and writing of the paper. SW: computational analysis. DY: TCR-transduced cell lines. All authors contributed to the article and approved the submitted version.

## Conflict of Interest

The authors declare that the research was conducted in the absence of any commercial or financial relationships that could be construed as a potential conflict of interest.

## Publisher's Note

All claims expressed in this article are solely those of the authors and do not necessarily represent those of their affiliated organizations, or those of the publisher, the editors and the reviewers. Any product that may be evaluated in this article, or claim that may be made by its manufacturer, is not guaranteed or endorsed by the publisher.

## Abbreviations

SMX: sulfamethoxazole
TCR: T cell receptor for antigen
TCC: T cell clones
MPE: maculopapular exanthema
SJS/TEN: Stevens-Johnson syndrome and toxic epidermal necrolysis.

## References

[1] Pichler WJ (editor). Drug Hypersensitivity. Karger (2007). 10.1159/isbn.978-3-318-01454-9

[2] Landsteiner K Jacobs J. Studies on the sensitization of animals with simple chemical compounds. J Exp Med. (1936) 64:625–39. 10.1084/jem.64.4.625 19870557 PMC2133443

[3] Park BK Naisbitt DJ Gordon SF Kitteringham NR Pirmohamed M. Metabolic activation in drug allergies. Toxicology. (2001) 158:11–23. 10.1016/S0300-483X(00)00397-8 11164988

[4] Martin SF. New concepts in cutaneous allergy. Contact Dermatitis. (2015) 72:2–10. 10.1111/cod.12311 25348820

[5] Cho T Uetrecht J. How reactive metabolites induce an immune response that sometimes leads to an idiosyncratic drug reaction. Chem Res Toxicol. (2017) 30:295–314. 10.1021/acs.chemrestox.6b00357 27775332

[6] Tailor A Meng X Adair K Farrell J Waddington JC Daly A . HLA DRB1^*^15:01-DQB1^*^06:02-restricted human CD4+ T cells are selectively activated with amoxicillin-peptide adducts. Toxicol Sci. (2020) 178:115–26. 10.1093/toxsci/kfaa128 32777075

[7] Stein P Weber M Prüfer S Schmid B Schmitt E Probst HC . Regulatory T cells and IL-10 independently counter regulate cytotoxic T lymphocyte responses induced by transcutaneous immunization. PLoS ONE. (2011) 6:e27911. 10.1371/journal.pone.0027911 22114725 PMC3218067

[8] Meng X Al-Attar Z Yaseen FS Jenkins R Earnshaw C Whitaker P . Definition of the nature and Hapten threshold of the β-lactam antigen required for T cell activation in vitro and in patients. J Immunol. (2017) 198:4217–7. 10.4049/jimmunol.1700209 28438900 PMC5444528

[9] Pichler WJ. Pharmacological interaction of drugs with antigen-specific immune receptors: the p-i concept. Curr Opin Allergy Clin Immunol. (2002) 2:301–5. 10.1097/00130832-200208000-00003 12130944

[10] Aster RH Curtis BR McFarland JG Bougie DW. Drug-induced immune thrombocytopenia: pathogenesis, diagnosis, and management. J Thromb Haemost. (2009) 7:911–8. 10.1111/j.1538-7836.2009.03360.x 19344362 PMC2935185

[11] Pichler WJ. Anaphylaxis to drugs: overcoming mast cell unresponsiveness by fake antigens. Allergy. (2020) 75:1340–9. 10.22541/au.158938598.83947005 32780486 PMC8247404

[12] Pichler WJ. The important role of non-covalent drug-protein interactions in drug hypersensitivity reactions. Allergy. (2021) 19:193–203. 34037262 10.1111/all.14962 PMC9291849

[13] Aster R. Drug induced thrombocytopenia. In: Platelets, Michelson A, editor. New York, NY: Academic press (2002). p. 593–606.

[14] Curtis BR. Drug-induced immune thrombocytopenia: incidence, clinical features, laboratory testing, and pathogenic mechanisms. Immunohematology. (2014) 30:55–65. 10.21307/immunohematology-2019-099 25247620

[15] Garratty G. Immune hemolytic anemia associated with drug therapy. Blood Rev. (2010) 24:143–50. 10.1016/j.blre.2010.06.004 20650555

[16] Pichler WJ Adam J Watkins S Wuillemin N Yun J Yerly D. Drug hypersensitivity: how drugs stimulate T cells via pharmacological interaction with immune receptors. Int Arch Allergy Immunol. (2015) 168:13–24. 10.1159/000441280 26524432

[17] Pichler WJ. Immune pathomechanism and classification of drug hypersensitivity. Allergy. (2019) 74:1457–71. 10.1111/all.13765 30843233

[18] Schnyder B Mauri-Hellweg D Zanni MP Bettens F Pichler WJ. Direct, MHC dependent presentation of the drug sulfamethoxazole to human αβ T cell clones. J Clin Invest. (1997) 100:136–41. 10.1172/JCI119505 9202065 PMC508173

[19] Zanni MP von Greyerz S Schnyder B Brander KA Frutig K Hari Y . HLA-restricted, processing- and metabolism-independent pathway of drug recognition by human ab T lymphocytes. J Clin Invest. (1998) 102:1591–8. 10.1172/JCI3544 9788973 PMC509010

[20] Greyerz S Zanni M Frutig K Schyder B Pichler WJ. Interaction of sulfonamide-derivatives with the TCR of sulfamethoxazole specific + T cell clones. J Immunol. (1999) 162:595–602. 9886437

[21] Pichler WJ Beeler A Keller M Lerch M Posadas S Schmid D . Pharmacological interaction of drugs with immune receptors: the p-i concept. Allergol Int. (2006) 55:17–25. 10.2332/allergolint.55.17 17075282

[22] Mallal S Nolan D Witt C Masel G Martin AM Moore C . Association between presence of HLA-B^*^5701, HLA-DR7, and HLA-DQ3 and hypersensitivity to HIV-1 reverse-transcriptase inhibitor abacavir. Lancet. (2002) 359:727–32. 10.1016/S0140-6736(02)07873-X 11888582

[23] Chung WH Hung SI Hong HS Hsih M-S Yang L-C Ho H-C . Medical genetics: a marker for Stevens-Johnson syndrome. Nature. (2004) 428:486. 10.1038/428486a 15057820

[24] Hung SI Chung WH Liou LB Chu CC Lin M Huang HP . HLA-B^*^5801 allele as a genetic marker for severe cutaneous adverse reactions caused by allopurinol. Proc Natl Acad Sci USA. (2005) 102:4134–9. 10.1073/pnas.0409500102 15743917 PMC554812

[25] Zhang FR Liu H Irwanto A Fu XA Li Y Yu GQ . HLA-B^*^13:01 and the dapsone hypersensitivity syndrome. N Engl J Med. (2013) 369:1620–8. 24152261 10.1056/NEJMoa1213096

[26] Konvinse KC Trubiano JA Pavlos R James I Shaffer CM Bejan CA . HLA-A^*^32:01 is strongly associated with vancomycin-induced drug reaction with eosinophilia and systemic symptoms. J Allergy Clin Immunol. (2019) 144:183–92. 10.1016/j.jaci.2019.01.045 30776417 PMC6612297

[27] Yun J Marcaida MJ Eriksson KK Jamin H Fontana S Pichler WJ . Oxypurinol directly and immediately activates the drug-specific T cells via the preferential use of HLA-B^*^58:01. J Immunol. (2014) 192:2984–93. 10.4049/jimmunol.1302306 24591375

[28] Mauri-Hellweg D Bettens F Mauri D Brander C Hunziker T Pichler WJ. Activation of drug-specific CD4+ and CD8+ T cells in individuals allergic to sulfonamides, phenytoin, and carbamazepine. J Immunol. (1995) 155:462–72. 7602118

[29] Deshpande P Hertzman RJ Palubinsky AM Giles JB Karnes JH Gibson A . Immunopharmacogenomics: mechanisms of HLA-associated drug reactions. Clin Pharmacol Ther. (2021) 110:607–15. 10.1002/cpt.2343 34143437 PMC8500648

[30] Schnyder B Burkhart CH Schnyder-Frutig K von Greyerz S Naisbitt D Pirmohamed M . Recognition of sulfamethoxazole and its reactive metabolites by drug specific T cells from allergic individuals. J Immunol. (2000) 164:6647–54. 10.4049/jimmunol.164.12.6647 10843725

[31] Nassif A Bensussan A Boumsell L Deniaud A Moslehi H Wolkenstein P . Toxic epidermal necrolysis: effector cells are drug-specific cytotoxic T cells. J Allergy Clin Immunol. (2004) 114:1209–15. 10.1016/j.jaci.2004.07.047 15536433

[32] Schmid DA Depta JP Pichler WJ. T cell-mediated hypersensitivity to quinolones: mechanisms and cross-reactivity. Clin Exp Allergy. (2006) 36:59–69. 10.1111/j.1365-2222.2006.02402.x 16393267

[33] Lerch M Keller M Britschgi M Kanny G Tache V Schmid DA . Cross-reactivity patterns of T cells specific for iodinated contrast media. J Allergy Clin Immunol. (2007) 119:1529–36. 10.1016/j.jaci.2007.02.007 17412404

[34] Britschgi M Steiner UC Schmid S Depta JP Senti G Bircher A . T-cell involvement in drug-induced acute generalized exanthematous pustulosis. J Clin Invest. (2001) 107:1433–41. 10.1172/JCI12118 11390425 PMC209321

[35] Meier-Schiesser B Feldmeyer L Jankovic D Mellett M Satoh TK Yerly D . Culprit drugs induce specific IL-36 overexpression in acute generalized exanthematous pustulosis. J Invest Dermatol. (2019) 139:848–58. 10.1016/j.jid.2018.10.023 30395846

[36] Vayne C Guéry EA Rollin J Baglo T Petermann R Gruel Y. Pathophysiology and diagnosis of drug-induced immune thrombocytopenia. J Clin Med. (2020) 9:2212. 10.3390/jcm9072212 32668640 PMC7408966

[37] Watkins S Pichler WJ. Sulfamethoxazole induces a switch mechanism in T cell receptors containing TCRVβ20-1, altering peptHLA recognition. PLoS ONE. (2013) 8:e76211. 10.1371/journal.pone.0076211 24116097 PMC3792127

[38] Watkins S Pichler Pichler WJ: Activating interactions of sulfanilamides with T cell receptors. Open J Immunol. (2013) 3:139–57. 10.4236/oji.2013.33019 PMC7613643 36172594

[39] Ko TM Chung WH Wei CY Shih HY Chen JK Lin CH . Shared and restricted T-cell receptor use is crucial for carbamazepine-induced Stevens-Johnson syndrome. J Allergy Clin Immunol. (2011) 128:1266–76.e11. 10.1016/j.jaci.2011.08.013 21924464

[40] Hammond S Thomson P Meng X Naisbitt D. *In-vitro* approaches to predict and study T-cell mediated hypersensitivity to drugs. Front Immunol. (2021) 12:630530. 10.3389/fimmu.2021.630530 33927714 PMC8076677

[41] Urbisch D Becker M Honarvar N Kolle SN Mehling A Teubner W . Assessment of pre- and pro-haptens using nonanimal test methods for skin sensitization. Chem Res Toxicol. (2016) 29:901–13. 10.1021/acs.chemrestox.6b00055 27070937

[42] Fentem J Malcomber I Maxwell G Westmoreland C. Upholding the EU's commitment to 'Animal Testing as a Last Resort' under REACH requires a paradigm shift in how we assess chemical safety to close the gap between regulatory testing and modern safety science. Altern Lab Anim. (2021) 2611929211040824. 10.1177/02611929211040824 34461762

[43] Adam J Wuillemin N Watkins S Jamin H Eriksson K Villiger P . Abacavir induced T cell reactivity represents an allo-immune reaction. PLoS ONE. (2014) 9:e95339. 10.1371/journal.pone.0095339 24751900 PMC3994040

[44] Lucas A Lucas M Strhyn A Keane NM McKinnon E Pavlos R . Abacavir-reactive memory T cells are present in drug naïve individuals. PLoS ONE. (2015) 10:e0117160. 10.1371/journal.pone.0117160 25674793 PMC4326126

[45] Engler OB Strasser I Naisbitt DJ Cerny A Pichler WJ. A chemically inert drug can stimulate T cells in vitro by their T cell receptor in non-sensitised individuals. Toxicology. (2004) 197:47–56. 10.1016/j.tox.2003.12.008 15003333

[46] Wuillemin N Adam J Fontana S Krähenbühl S Pichler WJ Yerly D. HLA haplotype determines hapten or p-i T cell reactivity to flucloxacillin. J Immunol. (2013) 190:4956–64. 10.4049/jimmunol.1202949 23596311

[47] Faulkner L Gibson A Sullivan A Tailor A Usui T Alfirevic A . Detection of primary T cell responses to drugs and chemicals in HLA-Typed volunteers: implications for the prediction of drug immunogenicity. Toxicol Sci. (2016) 154:416–29. 10.1093/toxsci/kfw177 27637899

[48] Alzahrani A Ogese M Meng X Waddington JC Tailor A Farrell J . Dapsone and Nitroso dapsone activation of Naïve T-cells from healthy donors. Chem Res Toxicol. (2017) 30:2174–86. 10.1021/acs.chemrestox.7b00263 29045131

[49] Ogese MO Lister A Gardner J Meng X Alfirevic A Pirmohamed M . Deciphering adverse drug reactions: in vitro priming and characterization of vancomycin-specific T cells from healthy donors expressing HLA-A^*^32:01. Toxicol Sci. (2021) 183:139–53. 10.1093/toxsci/kfab084 34175955 PMC8404995

[50] Pichler WJ Srinoulprasert Y Yun J Hausmann O. Multiple drug hypersensitivity. Int Arch Allergy Immunol. (2017) 172:129–38. 10.1159/000458725 28315874 PMC5472211

[51] Pan RY Chu MT Wang CW Lee YS Lemonnier F Michels AW . Identification of drug-specific public TCR driving severe cutaneous adverse reactions. Nat Commun. (2019) 10:3569. 10.1038/s41467-019-11396-2 31395875 PMC6687717

[52] Depta JP Altznauer F Gamerdinger K Burkhart C Weltzien HU Pichler WJ. Drug interaction with T-cell receptors: T-cell receptor density determines degree of cross-reactivity. J Allergy Clin Immunol. (2004) 113:519–27. 10.1016/j.jaci.2003.11.030 15007356

[53] Schmid DA Depta JP Lüthi M Pichler WJ. Transfection of drug-specific T-cell receptors into hybridoma cells: tools to monitor drug interaction with T-cell receptors and evaluate cross-reactivity to related compounds. Mol Pharmacol. (2006) 70:356–65. 10.1124/mol.105.021576 16617162

[54] Keller M Lerch M Britschgi M Tâche V Gerber BO Lüthi M . Processing-dependent and -independent pathways for recognition of iodinated contrast media by specific human T cells. Clin Exp Allergy. (2010) 40:257–68. 10.1111/j.1365-2222.2009.03425.x 20030663

[55] Yerly D Pompeu YA Schutte RJ Eriksson KK Strhyn A Bracey AW . Structural elements recognized by abacavir-induced T cells. Int J Mol Sci. (2017) 18:1464. 10.3390/ijms18071464 28686208 PMC5535955

[56] Villani AP Rozieres A Bensaid B Eriksson KK Mosnier A Albert F Mutez V . Massive clonal expansion of polycytotoxic skin and blood CD8^+^ T cells in patients with toxic epidermal necrolysis. Sci Adv. (2021) 7:eabe0013. 10.1126/sciadv.abe0013 33741590 PMC7978430

[57] Illing P Vivian JP Dudek NL Kostenko L Chen Z Bharadwaj M . Immune self-reactivity triggered by drug-modified HLA-peptide repertoire. Nature. (2012) 486:554–8. 10.1038/nature11147 22722860

[58] Zhou P Zhang S Wang Y Yang C Huang J. Structural modeling of HLA-B^*^1502/peptide/ carbamazepine/T-cell receptor complex architecture: implication for the molecular mechanism of carbamazepine-induced Stevens-Johnson syndrome/toxic epidermal necrolysis. J Biomol Struct Dyn. (2016) 34:1806–17. 10.1080/07391102.2015.1092476 26488421

[59] Cardone M Garcia K Tilahun ME Boyd LF Gebreyohannes S Yano M . A transgenic mouse model for HLA-B^*^57:01-linked abacavir drug tolerance and reactivity. J Clin Invest. (2018) 128:2819–32. 10.1172/JCI99321 29782330 PMC6025983

[60] Mallal S Phillips E Carosi G Molina JM Workman C Tomazic J . HLA-B^*^5701 screening for hypersensitivity to abacavir. N Engl J Med. (2008) 358:568–79. 10.1056/NEJMoa0706135 18256392

[61] Schnyder B Adam J Rauch A Thurnheer MC Pichler WJ. HLA-B^*^57:01(+) abacavir-naive individuals have specific T cells but no patch test reactivity. J Allergy Clin Immunol. (2013) 132:756–8. 10.1016/j.jaci.2013.04.013 23706613

[62] Rawlins MD Thompson JW. Pathogenesis of adverse drug reactions. In: Davies DM, editor. Textjournal of Adverse Drug Reactions. Oxford: Oxford University Press (1977). p. 10.

[63] Chen P Lin JJ Lu CS Ong CT Hsieh PF Yang CC . Carbamazepine-induced toxic effects and HLA-B^*^1502 screening in Taiwan. N Engl J Med. (2011) 364:1126–33. 10.1056/NEJMoa1009717 21428768

